# Correction to “Interplay of Local pH and Cation Hydrolysis during Electrochemical CO_2_ Reduction Visualized by In Operando Chemical Shift-Resolved Magnetic Resonance Imaging”

**DOI:** 10.1021/acs.jpcc.4c04245

**Published:** 2024-07-08

**Authors:** Michael Schatz, Johannes F. Kochs, Sven Jovanovic, Rüdiger-A. Eichel, Josef Granwehr

The original version of the article with DOI 10.1021/acs.jpcc.3c03563^1^ does not contain a data availability statement, which is supplied hereafter.

The NMR raw data acquired with the Bruker software TopSpin and the electrochemical data of electrolysis experiments acquired with the BioLogic software EC-Lab are available on the repository Jülich DATA at 10.26165/JUELICH-DATA/DNBEIN.^2^ Other data are provided by the authors upon request.
